# Movement Disorders in Neuromyelitis Optica Spectrum Disorder: A Systematic Review

**DOI:** 10.1002/mdc3.70339

**Published:** 2025-09-02

**Authors:** Luciana A.F. Bringel, Pedro L.G.S.B. Lima, Pedro V.F. Rodrigues, Francisco L.H.B. Cavalcante, Amanda R. Pinheiro, Fabricia M.S. Saraiva, Cynthia P. Rodrigues, Paulo V.S. Gonçalves, Leonardo J.R.A. Melo, Antônio E. Camelo‐Filho, Avelino M. Dutra‐Junior, Samuel R.O. Veras, Fernanda M.M. Carvalho, Flávia P.S. Rolim, Gabriela J. Martins, José A.C. D'Almeida, Pedro Braga‐Neto, Milena S. Pitombeira, Paulo R. Nobrega

**Affiliations:** ^1^ Division of Neurology Hospital Universitário Walter Cantídio, Universidade Federal do Ceará Fortaleza Brazil; ^2^ Graduate Program in Medical Sciences, University of Fortaleza Fortaleza Brazil; ^3^ Division of Neurology Hospital Geral de Fortaleza Fortaleza Brazil; ^4^ Campus Parque Ecológico, Centro Universitário Christus Fortaleza Brazil

**Keywords:** ataxia, dystonia, demyelinating autoimmune diseases, neuromyelitis optica, tremor

## Abstract

**Background:**

Several movement disorders (MD) have been reported to occur in neuromyelitis optica spectrum disorder (NMOSD). No extensive review has addressed the whole spectrum of MD in NMOSD.

**Objective:**

This article aims to review MD in NMOSD, describing its prevalence and features.

**Methods:**

A systematic review and prevalence meta‐analysis were conducted according to the Preferred Reporting Items for Systematic Reviews and Meta‐Analyses (PRISMA) reporting guidelines. We included articles on NMOSD patients with MD as defined by the 2015 international consensus criteria. Meta‐analysis was considered feasible if the prevalence of certain MDs was evaluated for at least 4 studies with more than 5 patients.

**Results:**

Ninety‐six articles were selected from an initial pool of 5441, involving 1751 patients, of whom 487 had MD. The prevalence of anti‐aquaporin‐4 antibodies (AQP4‐IgG) was 79.9% in general and 78.7% in NMOSD‐MD patients. Tonic spasms/paroxysmal dystonia and ataxia were the most prevalent MD in NMOSD, with 39% and 26% prevalence, respectively. Tremor, parkinsonism, myoclonus, chorea, and other hyperkinetic disorders were more rarely reported.

**Conclusions:**

A wide range of MDs in NMOSD were found, each with distinct features and frequency in literature. This knowledge might help to identify patients with NMOSD, which presents MD as a clinical feature and improves outcomes.

Neuromyelitis optica spectrum disorder (NMOSD), formerly known as Devic's disease, is 1 of the 2 most important acquired demyelinating diseases (DD) of the central nervous system (CNS), alongside multiple sclerosis (MS). In Latin American cohorts, the disease accounts for 11% to 18% of all cases of DDs, a much higher number than that in the European and North American series.^1^, ^2^, ^3^ NMOSD has a relapsing course, occurring in acute inflammatory attacks that cause severe CNS damage and produce long‐term neurological deficits.^4^, ^5^ The most common sites affected by the disease are the spinal cord and optic nerves, with area postrema and diencephalic lesions also occurring in a high proportion of patients.^5^

Demyelination‐related movement disorders (MD) encompass a spectrum of neurological manifestations secondary to inflammatory CNS diseases, particularly MS, NMOSD, and myelin oligodendrocyte glycoprotein antibody‐associated disease. Of these, MS is the most frequently implicated disorder, with reported movement abnormalities such as tonic spasms, restless legs syndrome (RLS), tremor, ataxia, parkinsonism, paroxysmal dyskinesias, dystonia, chorea, ballism, facial myoclonus such as hemifacial spasm and spastic paretic hemifacial contracture, and tics or tourettism.^5^, ^6^ These diverse manifestations typically result from direct demyelinating damage to specific pathways within the brain, brainstem, cerebellum, or spinal cord.^5^

Spinal‐generated movement disorders (SGMD) refer to those MDs in which the spinal cord plays an important role. The most accepted mechanism for SGMDs in DD is ephaptic transmission between partially demyelinated axons in the CNS,^7^ but alteration in supraspinal control leading to reduction in inhibitory spinal interneuron output and hyperexcitation of α‐motor neurons may also play a role in the genesis of this phenomenon.^8^ Patients with DD are an ideal population to study SGMDs due to the possibility of characterizing the exact anatomical generators of the movement through imaging of the spinal cord showing well‐defined lesion locations.

Previous studies have characterized MDs in MS and found that SGMDs were the most common manifestation in this population; however, patients with MS may also present abnormal movements related to basal ganglia involvement.^9^ In contrast, NMOSD presents a unique opportunity to study spinal MDs in DD, because most patients have no or minimal brain lesions and no neurodegeneration. This distinct pathological profile positions NMOSD as an optimal model to isolate and evaluate the SGMDs present in CNS inflammatory conditions.

Although there is also a substantial amount of literature reporting MD in NMOSD, to the best of our knowledge, there is no single large study reviewing the current literature regarding the spectrum of MD in NMOSD. We aim to describe the distinct MDs associated with NMOSD and characterize their unique features, including an epidemiological profile of the patients and topographic aspects of the demyelinating lesions.

## Patients and Methods

### Search Strategy and Selection Criteria

This systematic review was conducted according to the Preferred Reporting Items for Systematic Reviews and Meta‐Analyses (PRISMA) reporting guideline.^10^ A systematic search in PubMed/Medline, Scopus, Embase, and Web of Science databases was initially conducted from disease description in 1894^11^ to May 22, 2024. The terms and algorithms used in the search are summarized in Supporting Data S1. Rayyan app was utilized to organize and label the articles for peer analysis.^12^ Two independent researchers (P.V.F.R. and P.V.S.G.) evaluated the initial results for duplicate exclusion.

We included studies involving patients diagnosed with NMOSD who presented with MD during the disease course. NMOSD was defined according to the international consensus diagnostic criteria of 2015.^13^ There was no restriction regarding age, previous diseases, gender, or ethnicity.

MDs were defined as any excess or paucity of voluntary and automatic movements, unrelated to weakness or spasticity. Such a definition was initially introduced by Fahn and later classified in the same year.^14^, ^15^ This definition includes ataxia as a type of hyperkinetic MD. The terms “spasm,” “tonic spasm,” “paroxysmal dystonia,” and “tonic seizures” were frequently used interchangeably in the literature to describe alterations in muscle tone that produced stereotypical, recurrent, and localized movement lasting from seconds to a few minutes without evidence of consciousness impairment or epileptic activity. However, recent studies have better classified these paroxysmal abnormalities of muscle tone into either tonic spasms or paroxysmal dystonia. By applying the classification from Abboud and colleagues,^9^ we tried to classify these movement phenomena based on the information provided by the authors of each paper whenever possible.

Criteria for inclusion were (1) articles involving NMOSD patients presenting with MD; (2) articles with NMOSD defined as the 2015 International Panel for NMO Diagnosis; (3) articles published from inception to July 17, 2024; and (4) articles in English. Exclusion criteria were (1) reviews or conference abstracts, (2) articles including only other DDs, (3) articles that did not include any MD, (4) articles involving MDs related to co‐occurring disorders, (5) articles not accessible, and (6) articles involving only animal or cell models.

Two independent evaluators initially screened the studies from their titles and abstracts (F.L.H.B.C. and F.M.S.S). In cases of discordance, a third reviewer made the final decision (P.L.G.S.B.L.). Full texts of the resulting studies were evaluated according to eligibility criteria. Study selection results were reported using a PRISMA flowchart.

## Data Analysis

A standardized form was used for data extraction from included studies. Information extracted included country, author, year, objectives, study design, overall prevalence of MDs, prevalence of each form of MD, median age of subjects, sample size and type, time of MD presentation from NMOSD onset, and the instruments used for diagnosis.

The results were synthesized by organizing the findings of selected studies in search of patterns across them. After this initial step, possible explanations for data patterns, such as study design or sample characteristics, were elaborated on and used to guide the search for similar results in the already‐selected studies.

Articles were subdivided into small and large case series. This division aimed to select articles for a possible prevalence meta‐analysis of MD in NMOSD patients. Small case series were defined as articles including up to 5 subjects. Meta‐analysis was considered feasible if the prevalence of a certain MD was evaluated for at least 4 large studies.

## Results

**FIGURE 1:**
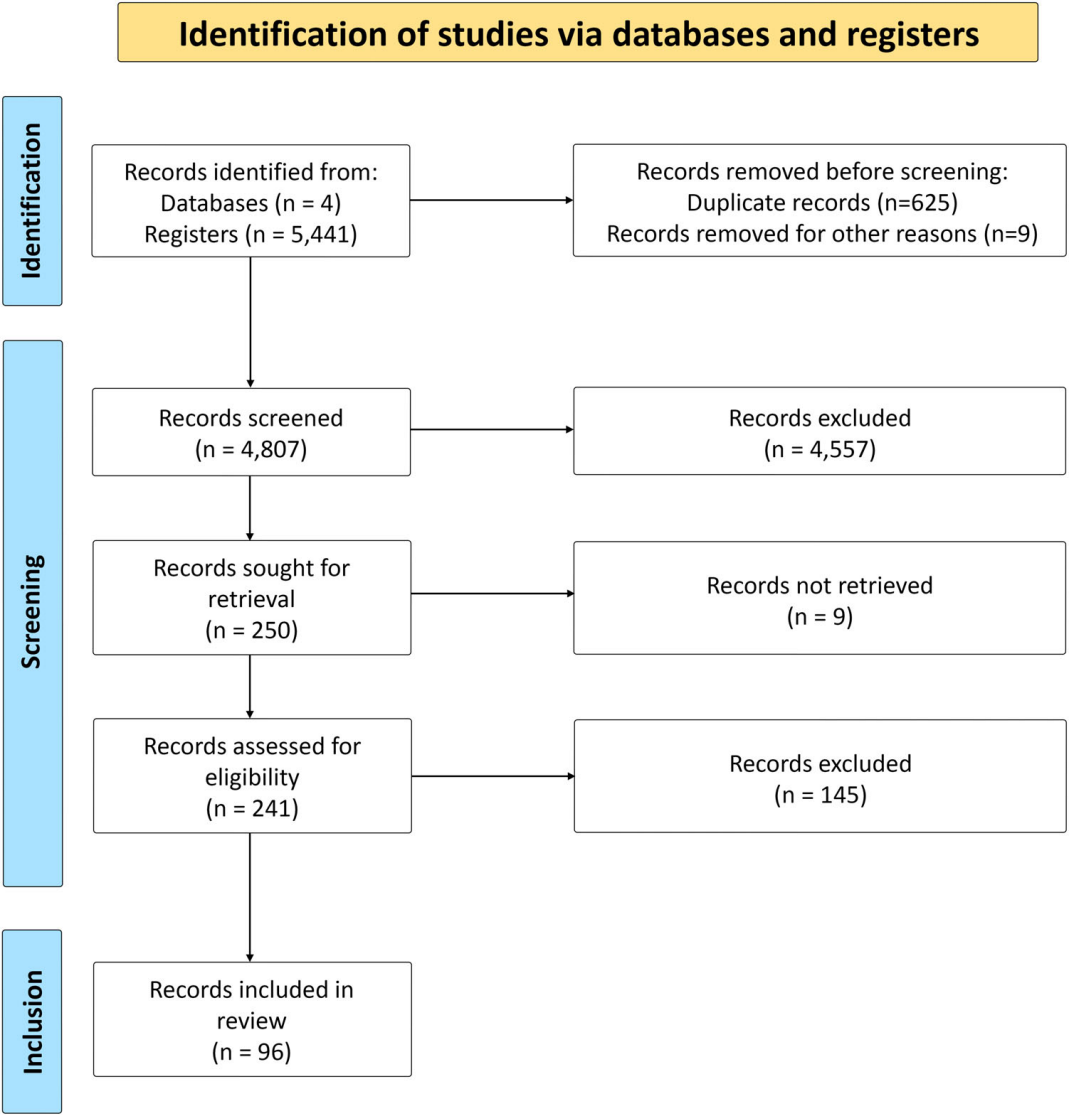
Flowchart showing the complete article selection procedures.

The initial literature search yielded 5441 articles. The first evaluation identified and removed 625 duplicates. Title and abstract screening identified 250 articles for full‐text analysis. Nine articles could not be retrieved and were excluded. Ninety‐six unique articles were included in the final review.^5^, ^9^, ^16^, ^17^, ^18^, ^19^, ^20^, ^21^, ^22^, ^23^, ^24^, ^25^, ^26^, ^27^, ^28^, ^29^, ^30^, ^31^, ^32^, ^33^, ^34^, ^35^, ^36^, ^37^, ^38^, ^39^, ^40^, ^41^, ^42^, ^43^, ^44^, ^45^, ^46^, ^47^, ^48^, ^49^, ^50^, ^51^, ^52^, ^53^, ^54^, ^55^, ^56^, ^57^, ^58^, ^59^, ^60^, ^61^, ^62^, ^63^, ^64^, ^65^, ^66^, ^67^, ^68^, ^69^, ^70^, ^71^, ^72^, ^73^, ^74^, ^75^, ^76^, ^77^, ^78^, ^79^, ^80^, ^81^, ^82^, ^83^, ^84^, ^85^, ^86^, ^87^, ^88^, ^89^, ^90^, ^91^, ^92^, ^93^, ^94^, ^95^, ^96^, ^97^, ^98^, ^99^, ^100^, ^101^, ^102^, ^103^, ^104^, ^105^, ^106^, ^107^, ^108^, ^109^ A complete flowchart of article selection procedures is shown in Figure 1.

Most articles included in the final analysis were small case series and case reports (n = 77/96, 80.2%).^17^, ^18^, ^19^, ^21^, ^23^, ^25^, ^26^, ^27^, ^28^, ^29^, ^30^, ^31^, ^33^, ^34^, ^35^, ^37^, ^38^, ^39^, ^40^, ^41^, ^42^, ^46^, ^47^, ^48^, ^49^, ^50^, ^51^, ^52^, ^53^, ^54^, ^55^, ^56^, ^57^, ^59^, ^60^, ^61^, ^62^, ^63^, ^64^, ^65^, ^66^, ^67^, ^68^, ^69^, ^70^, ^71^, ^72^, ^75^, ^76^, ^77^, ^78^, ^79^, ^80^, ^81^, ^82^, ^83^, ^84^, ^85^, ^86^, ^87^, ^88^, ^89^, ^90^, ^91^, ^93^, ^95^, ^96^, ^97^, ^98^, ^99^, ^100^, ^105^, ^106^, ^107^, ^108^, ^109^, ^110^ The studies included 1761 patients, of whom 487 presented with MD. Serological status was accessible for 1446 patients (82%), revealing a prevalence of AQP4‐IgG antibodies in 79.9% (n = 1156/1446) of the participants in general and in 78.7% (n = 240/314) of the NMOSD‐MD patients. Several distinct MDs were observed, with no superposition of MDs. Tonic spasms and ataxia were the most prevalent MDs reported in NMOSD.

**FIGURE 2:**
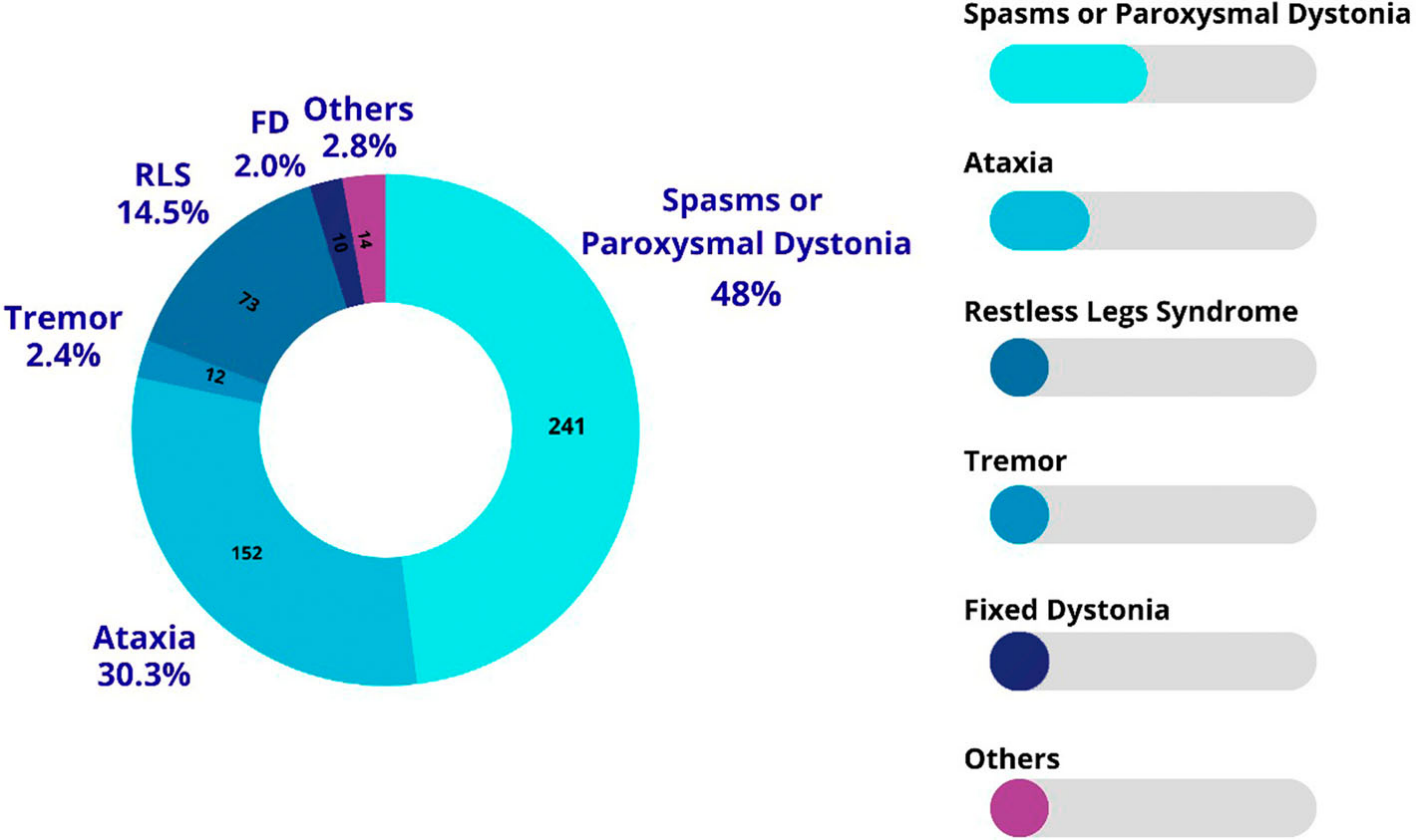
Frequency of movement disorders reported in neuromyelitis optica spectrum disorder. FD, fixed dystonia. RLS, restless legs syndrome.

Only 1 large study has evaluated the global prevalence of MDs in general in NMOSD patients.^5^ da Silva and colleagues recruited 47 adult NMOSD patients to be evaluated by an MDs specialist. They found that 38.3% (n = 18/47) of the patients had some MD.^5^ Two other significantly large studies have also evaluated MD in NMOSD, using a slightly different methodology.^9^, ^16^ Abboud et al. in 2016 and Abboud et al. in 2024 prospectively studied NMOSD patients to evaluate the presence of SGMDs, excluding ataxias and other traditionally encephalic MD.^9^, ^16^ The first study with 37 patients found a prevalence of 43.2% (n = 16/37), and the second study registered an even higher prevalence, observing SGMDs in 21 of 25 patients (84%). A fourth large study evaluated self‐reported symptoms in 140 NMOSD patients through a standardized questionnaire and found that at least 34% (n = 48/140) of this population had MD.^102^ Table 1 summarizes the main prevalence studies included and reviewed, and Figure 2 summarizes the frequency of the MD reported. The prevalence and characteristics of each MD in the disease are discussed here.

**TABLE 1:** Summary of the main prevalence studies

Study	Year	Study type	Population	Number of patients	Seropositivity	Number with MD	MD evaluated
Usmani et al.^32^	2012	Retrospective	United States	57	NA	8	Spasm
Abaroa et al.^94^	2013	Retrospective	Argentina	22	NA	18	Spasm
Cheng et al.^44^	2013	Retrospective	China	106	85% (n = 90/106)	9	Ataxia
Kremer et al.^36^	2014	Prospective	Cross‐populational*	258	79.4% (n = 205/258)	1	Ataxia
Lin et al.^43^	2014	Retrospective	China	57	29.8% (n = 17/57)	11	Ataxia
Abboud et al.^9^	2016	Retrospective	United States	37	91.9% (n = 34/37)	16	Dystonia Spasm Tremor Myoclonus
Hyun et al.^58^	2016	Prospective	Korea	153	90% (n = 137/153)	23	RLS
Liu et al.^74^	2017	Prospective	China	230	78.7% (n = 181/230)	52	Spasm
da Silva et al.^5^	2018	Prospective	Brazil	47	63.9% (n = 30/47)	18	Dystonia Spasm Tremor RLS
Li et al.^73^	2020	Retrospective	China	153	83.7% (n = 128/153)	67	Spasm
Shaygannejad et al.^45^	2020	Prospective	Iran	24	NA	11	RLS
Chang et al.^111^	2021	Retrospective	China	129	56.6% (n = 73/129)	22	Tremor Ataxia
Campetella et al.^24^	2023	Retrospective and prospective	Italy	27	100% (n = 27/27)	11	Spasm
Abboud et al.^16^	2024	Prospective	United States	25	48% (n = 12/25)	22	Dystonia Spasm Tremor Myoclonus RLS
Osborne et al.^104^	2024	Retrospective	United States	14	92.9% (n = 13/14)	4	Spasm
Dinoto et al.^103^	2025	Retrospective	United States	16	100% (n = 16/16)	10	Spasm Tremor
Liang et al.^102^	2025	Prospective	China	140	N/A	48	Ataxia
Ozdogar et al.^101^	2025	Prospective	Turkey	56	N/A	34	RLS

Abbreviations: MD, movement disorder; NA, not available; RLS, restless legs syndrome. * France, United States, United Kingdom, Japan, Germany.

### Spasm/Paroxysmal Dystonia/Tonic Seizures

Tonic spasm and paroxysmal dystonia are the MDs most commonly associated with NMOSD, and they are the subjects of the largest studies reviewed. Thirty‐one studies involving 241 participants described patients with spasms or paroxysmal dystonia in the setting of NMOSD.^5^, ^9^, ^16^, ^17^, ^24^, ^28^, ^32^, ^37^, ^47^, ^48^, ^53^, ^60^, ^61^, ^62^, ^67^, ^73^, ^74^, ^75^, ^76^, ^79^, ^80^, ^81^, ^87^, ^90^, ^93^, ^94^, ^104^, ^106^, ^108^, ^109^

**FIGURE 3:**
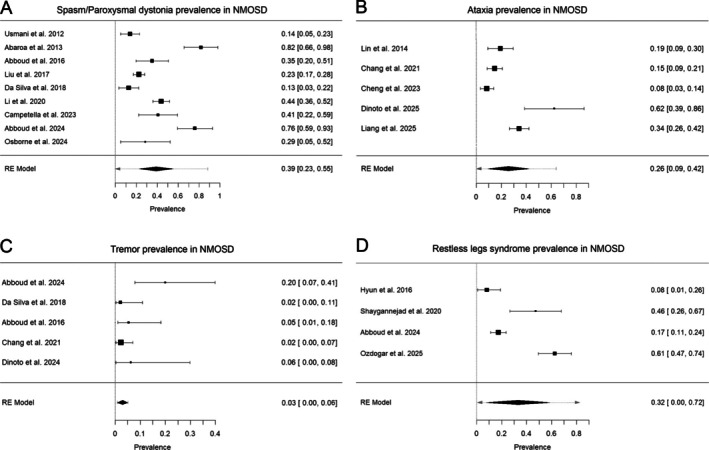
Forest plots showing the estimated prevalence of (**A**) tonic spasms, (**B**) ataxia, (**C**) tremor, and (**D**) restless legs syndrome in NMOSD among studies. NMOSD, neuromyelitis optica spectrum disorder.

Nine large case series evaluated spasms in NMOSD.^5^, ^9^, ^16^, ^24^, ^32^, ^73^, ^74^, ^94^, ^104^ These studies included 210 patients from a representative population of NMOSD. Their clear diagnostic and outcome definitions allowed us to perform a meta‐analysis of the prevalence of spasms in NMOSD assessment. The cumulative prevalence of either tonic spasm or paroxysmal dystonia in NMOSD populations studied across different reports was 39% (95% confidence interval [CI] from 22.8% to 55.2%). However, there was high heterogeneity among studies, with an I^2^ of 95%. Figure 3A shows a complete view of the studies in the analysis, their effects, and population scores.

The reclassification of MDs led to the identification of a high proportion of tonic spasms compared to paroxysmal dystonia (185 cases of tonic spasms vs. 56 cases of paroxysmal dystonia).

Patients with paroxysmal events had an average of 45.9 years at MD presentation. Only 1 pediatric patient was reported, presenting spasms by the age of 2.^28^ No significant difference in age at disorder presentation was observed between spasm and paroxysmal dystonia subgroups, with the tonic spasm group presenting a mean age of 46.46 years and the Parkinson's disease group of 43.7 years. Ninety‐three percent of the patients who had gender data were women (n = 150/160). Previous studies have reported a positive association between tonic spasms and anti‐AQP4 seropositivity.^2^, ^23^ In one previous study, patients with SGMDs presented at a significantly older age and were more frequently Caucasian or Hispanic compared to those without spinal movement disorders (SMDs).^9^

Disease duration by the time of spasm presentation could be retrieved in 77.6% of the patients (n = 187/241). Spasms appear to occur at any time in the disease course, with isolated studies reporting a high variability regarding the time to MD presentation. The occurrence of spasms at disease onset was uncommon in literature, observed only in 12 patients (6.4%). We observed a significant delay in the onset of spasms in patients who did not present with MD during the initial attack of the disease, with spasms developing after an average of 12 years from diagnosis.^24^

A total of 178 patients had the body segment of the events described. Paroxysmal events affecting the lower extremities were the most frequent, either isolated (n = 65/178, 36.5%) or in association with the upper extremities (n = 37/178, 20.8%). MDs affecting all 4 extremities and only in the upper extremities were also common, occurring in 15.7% (n = 28/178) and 14.6% (n = 26/178) of patients, respectively. The trunk was less affected by spasms or paroxysmal dystonia, observed in 12 patients associated with extremity events (6.8%) and in 8 patients in isolation (4.5%). Neck spasms were observed only in 2 patients (1.1%). No association was observed between the body segment and the type of paroxysmal event.

Even though paroxysmal events are not universally painful, the majority of patients report pain that is associated with significant quality‐of‐life impairment related to predisposition to falls, limitations in activities of daily living, and increased use of the health‐care system.^112^ All patients who presented spasms or paroxysmal dystonia and had data regarding the topography of their lesions had cervical and/or thoracic spine involvement, isolated or in association with other CNS lesions. No other lesion site was consistently reported to be related to the occurrence of paroxysmal events.

These observations confirm spasms and paroxysmal dystonia as common MDs in NMOSD, occurring in almost half of the patients. Although more recent studies show a consistently higher prevalence of tonic spasms in progressive MS,^113^ their prevalence in MS seems to be lower compared to NMOSD, being observed in 3.8% to 39.4% of the patients.^94^, ^113^ This significant difference may reflect their distinct pathogenic mechanisms. Unlike MS, where oligodendrocyte damage drives demyelination, NMOSD is an anti‐AQP4‐mediated astrocytopathy, with secondary demyelination and extensive damage particularly in the spinal cord.^114^

Although tonic spasms in NMOSD are considered a typical SGMD, they do not exclusively occur in the acute phase of a transverse myelitis episode. Kim et al.^115^ have reported a mean latency of 48 days after the initial myelitis attack, in which the episodes were not accompanied by new neurological symptoms or new lesions on magnetic resonance imaging (MRI). Additionally, in this cohort, the length of the spinal cord damage and the presence of lesions on brain MRI were not significantly associated with the appearance of tonic spasms. The authors hypothesized that the occurrence during the recovery phase may be linked to partial remyelination of the spinal cord rather than the demyelination itself.^115^ Considering that tonic spasms can also occur in the acute phase of short transverse myelitis, as shown by Flanagan et al., and due to the classical central pattern of spinal cord lesions in NMOSD, considerations can be made about the role of early disruption of centripetal fibers in the spinal cord when the corticospinal fibers were relatively intact.^116^

### Ataxia

Ataxia was the second most common MD related to NMOSD and the most cited by individual articles. A total of 56 unique articles described 152 patients who developed ataxia during the disease course.^18^, ^19^, ^20^, ^21^, ^23^, ^25^, ^26^, ^27^, ^29^, ^30^, ^31^, ^33^, ^35^, ^36^, ^38^, ^40^, ^41^, ^42^, ^43^, ^44^, ^46^, ^50^, ^51^, ^54^, ^55^, ^56^, ^57^, ^59^, ^64^, ^65^, ^66^, ^68^, ^69^, ^71^, ^72^, ^77^, ^80^, ^82^, ^83^, ^84^, ^85^, ^86^, ^89^, ^91^, ^95^, ^96^, ^97^, ^100^, ^103^, ^105^, ^107^, ^110^, ^111^ Six major studies reported ataxia in NMOSD patients. These studies reported a pooled prevalence of 26% (95% CI 9%–42%). High heterogeneity was observed among studies (I^2^ of 95%). One of the studies reported a much lower prevalence compared to others (0.77%).^36^ However, this study used a methodology different from other observational studies, focusing on brainstem signs and symptoms in NMOSD, and data were collected using standardized forms to achieve this objective. This may have lead to omission of other signs and symptoms that may characterize ataxia. This approach was considered substantially distinct from others, and the study was not considered eligible for meta‐analysis or incidence estimation. Figure 3B shows the included studies in the analysis and the pooled prevalence.

The mean age of patients with ataxia was 37.5 years, and most of the patients were women (n = 41/50). Ataxia was most commonly observed at disease presentation, occurring in 80% (n = 36/45) as a symptom of onset. In patients who did not present with ataxia as the first symptom, this symptom occurred on average 2.13 years after the initial presentation, ranging from 1 month to 10 years. No specific pattern of ataxia was consistently reported among the reviewed studies. Most of the patients perceived ataxia as an impairment in gait, whereas both truncal and limb ataxia were observed in most specialist evaluations.

In reviewed articles spinal cord lesions were the most frequent site of injury described in patients with NMOSD presenting with ataxia, occurring in 22 patients (42.3%). Most of these patients presented with cervical and upper thoracic lesions. No lumbar spinal cord lesions were described in the ataxia subpopulation. Other sites of lesions frequently reported were medulla oblongata (n = 20/52, 38.4%), cerebellar peduncles (n = 9/52, 17.3%), and diencephalon (n = 8/52, 15.4%). Corpus callosum, cerebellar hemispheres, corona radiata, periaqueductal gray matter, and cerebral hemispheric lesions were also reported.

A systematic reclassification of ataxia into cerebellar versus sensory subtypes was not possible due to insufficient data on the specific characteristics of the MDs. We hypothesize that the majority of cases were sensory ataxia secondary to spinal cord lesions. This interpretation is supported by the finding that nearly half of the patients with ataxia had documented spinal cord abnormalities on MRI. Moreover, the number of patients with spinal lesions may be even greater, as low‐resolution imaging could have failed to detect subtle abnormalities.

The term “ataxia” could have been used incorrectly in some of the reviewed articles to describe abnormal gait due to weakness. This may have led to an overestimation of the number of patients with ataxia.

### Tremor

Although tremor is reported to be the most frequent MD associated with MS, only 5 studies have reported patients with NMOSD‐associated tremor, usually affecting the upper limbs with predominant postural/kinetic features.^5^, ^9^, ^16^, ^103^, ^111^ These studies reported 12 patients, making tremor a rare presentation in these cohorts, with prevalence rates ranging from 2% to 20% between reports. A meta‐analysis of the published cohorts revealed a pooled prevalence of 3% (95% CI from 0% to 6%). There was substantial heterogeneity among studies, with an I^2^ of 41%. Figure 3C shows a complete view of the studies in the analysis, their effects, and population scores. The data reporting the site of the lesion in NMOSD‐associated tremor could not be reliably retrieved.

### Restless Legs Syndrome

Seventy‐three patients reported RLS associated with NMOSD, being described in 4 separate articles.^16^, ^45^, ^58^, ^101^ RLS was diagnosed in the studies based on the updated criteria set forth by the International Restless Legs Syndrome Study Group consensus. The diagnostic questionnaire incorporates 5 key characteristics: 4 reported by the patient and 1 involving a physician's evaluation to rule out other potential causes. The pooled prevalence of RLS in NMOSD was 32.2% (95% CI from 0% to 72%). Figure 3D shows study‐by‐study prevalence data and the pooled prevalence.

Ozdogar et al.^101^ were the only team to report the characteristics of patients with NMOSD and RLS. The mean age of these patients was 45.6 years (standard deviation 13 years), with most of them being women (85.3%, n = 29/34). They also observed a mean difference of 8.59 years between disease onset and the occurrence of RLS symptoms. Statistical significance was achieved when Expanded Disability Status Scale (EDSS) scores were compared in NMOSD patients with and without RLS, revealing that RLS‐positive patients had higher scores.

Hyun and colleagues enrolled 159 NMOSD patients and 153 age‐ and gender‐matched healthy controls to determine the relation between RLS and NMOSD.^58^ They found a prevalence of 17% of RLS in the NMOSD group compared to 7.8% in the control group (*P* = 0.015).^58^ Scores of RLS severity, such as the International RLS Severity Scale, Fatigue Severity Scale, and Pittsburgh Sleep Quality Index, were also significantly higher in the NMOSD group.^58^ They documented that 88.5% of their cohort of patients with NMOSD‐associated RLS (n = 23/26) developed RLS at or after the onset of NMOSD.^58^

The study by Shaygannejad et al. found the highest prevalence of RLS in NMOSD, with an estimated prevalence of 45.8%.^45^ Their cohort compared patients with NMOSD‐associated RLS with a group with MS‐associated RLS.^45^ Despite the high prevalence of RLS in NMOSD in the cohort, they found that RLS was significantly higher in individuals with MS, especially secondary progressive MS.^45^

Only 1 study assessed the site of lesions in patients with NMOSD‐associated RLS. Ozdogar et al.^101^ reported that cervical spinal cord lesions were present in 43.8% (n = 14/32) of patients, whereas thoracic spinal cord lesions were observed in 62.5% (n = 20/32). Other lesion sites or patterns of lesion occurrence (whether isolated or in association) were not specified.

### Dystonia

Two studies that comprised 10 NMOSD patients described nonparoxysmal focal dystonia.^9^, ^16^ This phenomenon differentiates itself from paroxysmal dystonia or tonic spasm by sustained agonist–antagonist simultaneous muscle contraction. Unlike paroxysmal dystonia, the fixed dystonia presented by some patients is reported to be dissociated from pain.^9^ The study by Abboud et al.^16^ found that focal dystonia is one of the few MDs in NMOSD that does not have an intermittent pattern and seems to occur at a similar rate among NMOSD and other demyelinating disorders.^16^

Four studies reported the site of lesion in NMOSD‐associated dystonia, including 8 patients. All patients had lesions restricted to the spinal cord.^9^, ^61^, ^79^, ^87^ Isolated cervical spinal cord lesions were the most frequently found, observed in 62.2% (n = 5/8) of these patients. Combined cervical and thoracic lesions were observed in 2 patients. An isolated thoracic lesion was reported in 1 patient. No lumbar or supraspinal lesions were reported in the dystonia NMOSD subpopulation.

### Other Movement Disorders

Myoclonus is a rare NMOSD presentation in literature, reported in 6 patients by 3 different articles.^16^, ^34^, ^88^ Two of these articles were case reports, and 1 was a larger cohort of NMOSD patients.^16^, ^34^, ^88^ In all patients, myoclonus was reported to affect only 1 body segment. One patient was reported to elicit the jerks under auditory stimuli and glabella tapping.^34^ A pharmacologic approach with baclofen or a combination of clonazepam and levetiracetam ameliorated the myoclonic jerks in some reports.

Two patients with NMOSD developed parkinsonism.^49^, ^52^ The patients had strategic basal ganglia lesions and developed cogwheel rigidity and bradykinesia. Interestingly, both had mental status alteration associated with the onset of parkinsonism, characterized by hypersomnolence and cognitive decline. Only 1 patient had parkinsonism as the first presentation of NMOSD.^49^

Chorea occurred in 2 patients in the literature.^39^, ^78^ Both patients presented with involuntary choreoathetoid movements in a single arm. Hyperintensities in the cervical spinal cord were the only identifiable demyelinating lesions in these patients. One of the patients had a positive temporal correlation between the introduction of gabapentin for tonic spasms and the appearance of chorea.^39^ In both cases, choreic movements improved after immunotherapy and did not recur at the last follow‐up.

Two case reports described complex hyperkinetic disorders in the setting of NMOSD.^20^, ^46^ The movements were irregular, random, and nonrhythmic, and worsened with eye closure. Looking at the affected extremity greatly improved the movements. Both cases of complex hyperkinetic disorders coexisted with sensory ataxia.

Catatonia was reported in 1 patient.^50^, ^63^ The patient reported by Alam et al. developed a catatonic state associated with nihilistic delusions in the first year after the initial NMOSD attack.^63^ This manifestation was related to a large lesion within the right posterior frontal/anterior parietal/anterior temporal lobe.^63^ The patient improved after immunotherapy and antipsychotics.

Shozawa and colleagues reported a patient who had a strategic lesion in the corpus callosum, presenting with callosal disconnection syndromes, mainly diagonistic apraxia.^50^ She experienced abnormal movements in her left hand when doing usual tasks, like dressing and eating,^50^ suggesting an alien hand phenomenon. This patient improved after immunotherapy.

## Conclusion

Despite advances in characterizing MDs in demyelinating disorders, our study has significant limitations regarding the classification and subtyping of specific manifestations such as dystonia, spasms, and ataxia. Inconsistent terminology across studies (eg, overlapping use of “spams,” “dystonia,” or “hyperkinetic movements”) complicates comparative analyses, and some authors have grouped the MDs under broad phenotypic categories without distinguishing their underlying anatomical substrates and radiological correlates (eg, spinal vs. supraspinal). This lack of granularity precludes more specific analyses of MD subtypes—such as cerebellar versus sensory ataxia—which may have distinct prognostic or therapeutic implications.

Nevertheless, we systematically reviewed the existing literature on MDs in NMOSD, demonstrating their wide clinical spectrum. Tonic spasms and ataxia were the most common MDs. More studies are necessary with standardized phenotyping protocols and advanced neuroimaging correlations to better characterize the prevalence and pattern of each MD in NMOSD. These findings might help further characterize the clinical manifestations of NMOSD and identify diagnostic clues that may aid in clinical practice.

## Author Roles

(1) Research project: A. Conception, B. Organization, C. Execution; (2) Statistical analysis: A. Design, B. Execution, C. Review and critique; (3) Manuscript preparation: A. Writing of the first draft, B. Review and critique.

L.A.F.B.: 1A, 1B, 1C, 3A, 3B

P.L.G.S.B.L.: 1A, 1B, 1C, 2A, 2B, 2C, 3A, 3B

P.V.F.R.: 1B, 1C, 3A

F.L.H.B.C.: 1B, 1C, 3A

A.R.P.: 1B, 1C, 3A

F.M.S.S.: 1B, 1C, 3A

C.P.R.: 1B, 1C, 3A

P.V.S.G.: 1B, 1C, 3A

L.J.R.A.M.: 1B, 1C, 3A

A.E.C.‐F.: 1A, 1B, 1C, 3A

A.M.D.‐J.: 1A, 1B, 1C, 3A

S.R.O.V.: 1A, 1B, 1C, 3A

F.M.M.C.: 1A, 1B, 1C, 3A, 3B

F.P.S.R.: 1A, 1B, 1C, 3A, 3B

G.J.M.: 2B, 2C, 3A, 3B

J.A.C.D.: 2B, 2C, 3A, 3B

P.B.‐N.: 2B, 2C, 3A, 3B

M.S.P.: 2B, 2C, 3A, 3B

P.R.N.: 1A, 1B, 2A, 2C, 3A, 3B

## Disclosures

**Ethical Compliance Statement:** We confirm that we have read the journal's position on issues involved in ethical publication and affirm that this work is consistent with those guidelines. The authors confirm that the approval of an institutional review board was not required for this work. No informed consent was obtained for this review.

**Funding Sources and Conflicts of Interest:** No specific funding was received for this work. The authors declare that there are no conflicts of interest relevant to this work.

**Financial Disclosures for the Previous 12 Months:** The authors declare that there are no additional disclosures to report.

## Supporting information


**Supplementary Material S1** Terms and algorithms used in the article search.

## Data Availability

The data that support the findings of this study are available from the corresponding author upon reasonable request.
