# Supplementary material for: Movement Disorders in Neuromyelitis Optica Spectrum Disorder: A Systematic Review
Source: Mov Disord Clin Pract. 2025 Sep 2;13(3):600–10. doi: 10.1002/mdc3.70339 (PMC13042436; doi:10.1002/mdc3.70339)
Supplement: Supplementary file 1 — Supplementary Material S1 Terms and algorithms used in the article search. [file MDC3-13-600-s001.docx]

**Supplementary Material 1. Detailed search strategy for the systematic literature review**

**Search algorithm:**

("Neuromyelitis Optica Spectrum Disorder" OR "NMO Spectrum Disorder" OR "NMO" OR "Neuromyelitis Optica" OR "Devic's Disease" OR "AQP4+NMOSD" OR "AQP4") AND ("Movement Disorders" OR "Dyskinesias" OR "Dyskinesia" OR "Dyskinesia Syndromes" OR "Movement Disorder Syndrome" OR "Hyperkinesia" OR "Hyperkinetic" OR "Hyperkinetic Movement Disorder" OR "Abdominal dyskinesias" OR "Akathitic movements" OR "Akathitic" OR "Akathisia" OR "Ataxia" OR "Dyssynergia" OR "Coordination Impairment" OR "Ataxy" OR "Incoordination" OR "Asynergia" OR "Dysmetria" OR "Athetosis" OR "Athetoses" OR "Athetoid Movements" OR "Ballism" OR "Ballismus" OR "Hemiballismus" OR "Hemiballism" OR "Chorea" OR "Choreas" OR "Chorea Syndrome" OR "Dystonia" OR "Muscle Dystonia" OR "Dystonic" OR "Dystonic disorder" OR "Hemifacial spasm" OR "Facial Spasm" OR "Hyperekplexia" OR "Hypnogenic dyskinesias" OR "Jumping disorder" OR "Jumpy stumps" OR "Moving toes and fingers" OR "Myoclonus" OR "Myoclonic Jerk" OR "Myoclonic" OR "Myokymia" OR "synkinesia" OR "synkinesis" OR "Myorhythmia" OR "Paroxysmal dyskinesias" OR "Periodic movements in sleep" OR "Sleep Myoclonus Syndrome" OR "REM sleep behavior disorder" OR "REM Behavior Disorder" OR "Rapid Eye Movement Sleep Behavior Disorder" OR "Restless legs" OR "Restless Legs Syndrome" OR "Stereotypy" OR "Stereotypic Movement Disorders" OR "Tremor" OR "Tremors" OR "Tic" OR "Tics" OR "Tic disorder" OR "Hypokinesia" OR "Hypokinetic" OR "Hypokinetic Movement Disorder" OR "Akinesia" OR "Bradykinesia" OR "Bradykinetic" OR "Parkinsonian Disorder" OR "Parkinsonism" OR "Parkinsonian Syndrome" OR "Apraxia" OR "Dyspraxia" OR "Blocking tics" OR "Holding tics" OR "Tic" OR "Tics" OR "Tic disorder" OR "Motor tic" OR "Cataplexy" OR "Drop attack" OR "Drop attacks" OR "Cataleptic" OR "Cataleptic Attack" OR "Catatonia" OR "Psychomotor depression" OR "obsessional slowness" OR "Freezing phenomenon" OR "Freezing" OR "FOG" OR "Freezing of gait" OR "Hesitant gait" OR "Hesitant gaits" OR "Hypothyroid slowness" OR "Rigidity" OR "Muscle Rigidity" OR "Muscular Rigidity" OR "Cogwheel Rigidity" OR "Extrapyramidal Rigidity" OR "Stiff muscles" OR "Muscular Stiffness" OR "Muscle Stiffness")
